# Representations of time in human frontoparietal cortex

**DOI:** 10.1038/s42003-018-0243-z

**Published:** 2018-12-21

**Authors:** Masamichi J. Hayashi, Wietske van der Zwaag, Domenica Bueti, Ryota Kanai

**Affiliations:** 1 Global Center for Medical Engineering and Informatics, Osaka University, Suita, 565-0871 Japan; 2 School of Psychology, University of Sussex, Brighton, BN1 9QH UK; 3 Center for Biomedical Imaging, Ecole Polytechnique Fédérale de Lausanne, Lausanne, CH-1015 Switzerland; 4 Spinoza Centre for Neuroimaging, Amsterdam, 1105BK The Netherlands; 5 International School for Advanced Studies, Trieste, 34136 Italy; 6 Araya Inc., Tokyo, 105-0003 Japan; 7 Sackler Centre for Consciousness Science, University of Sussex, Brighton, BN1 9QH UK

**Keywords:** Perception, Human behaviour

## Abstract

Precise time estimation is crucial in perception, action and social interaction. Previous neuroimaging studies in humans indicate that perceptual timing tasks involve multiple brain regions; however, whether the representation of time is localized or distributed in the brain remains elusive. Using ultra-high-field functional magnetic resonance imaging combined with multivariate pattern analyses, we show that duration information is decoded in multiple brain areas, including the bilateral parietal cortex, right inferior frontal gyrus and, albeit less clearly, the medial frontal cortex. Individual differences in the duration judgment accuracy were positively correlated with the decoding accuracy of duration in the right parietal cortex, suggesting that individuals with a better timing performance represent duration information in a more distinctive manner. Our study demonstrates that although time representation is widely distributed across frontoparietal regions, neural populations in the right parietal cortex play a crucial role in time estimation.

## Abstract (web summary)

Masamichi Hayashi et al. combine high field neuroimaging (7T fMRI) and multivariate pattern analyses to show that the pattern of functional MRI activity in the right parietal lobe can predict the perception of time in individual participants. They find that while time representation is distributed across frontoparietal regions, the right parietal cortex plays a key role.

## Introduction

The ability to estimate time intervals in the range of hundreds of milliseconds is crucial in many aspects of our perception, action and social interaction, such as in playing music, dancing, speech perception, and generation^1,2^, as well as in simple tasks, such as turn taking^3^. Theoretical models of temporal processing have proposed various time representations^4^, such as neural oscillations (e.g., clock-counter model, beat-frequency model)^1,5^, or dynamics of neural activities in neural networks (e.g., state-dependent network model, population clock model)^6,7^. More recently, a duration-channel model proposed that time is represented by neural populations tuned to specific time intervals^8^. However, how these time representations are implemented in the human brain has remained unknown to date.

The key question in the neural implementation of a timing system is whether the time representation is localized in a specific part of the brain or distributed across the brain^9,10^. A previous meta-analysis of neuroimaging studies indicated that perceptual and motor timing tasks that involved the processing of sub-second time intervals activated multiple brain regions, such as the bilateral supplementary motor area (SMA), middle frontal gyrus, inferior parietal lobule (IPL), inferior frontal gyrus (IFG), and posterior cerebellum as well as the right basal ganglia and insula^11^. However, it has remained unclear whether these regions explicitly encode time information or simply reflect cognitive operations, such as attention or working memory processes that are necessary for time estimation tasks^5,12,13^.

To date, there is only limited evidence of the existence of explicit time representations in humans^14^. Using a functional magnetic resonance imaging (fMRI) adaptation technique^15,16^, we previously identified a reduction in the blood oxygenation level-dependent (BOLD) response when the stimuli of the same duration were repeated (i.e., repetition suppression), which suggests the existence of duration-tuned neural populations in the right IPL^14^. This finding provides evidence of duration channels, which were proposed by a previous psychophysical study showing repellent-type behavioral aftereffects following psychophysical adaptation to a specific duration^8,17–21^. Although the fMRI adaptation technique has been widely used to identify the locus of explicit neural representation for various stimulus features, repetition suppression is an indirect measure of assessing the existence of explicit neural representation, and the exact physiological mechanism of repetition suppression remains a matter of debate^15,22^. By contrast, multivariate pattern analysis (MVPA) directly captures small biases in spatial activity patterns produced by feature selective neural populations^23,24^ and is thus particularly suitable for the identification of brain regions that carry duration information.

To determine whether the brain regions previously implicated in time perception represent time directly, we designed an fMRI experiment for a region of interest (ROI)-based MVPA, which allowed us to determine which brain areas carry duration information. To provide complementary information to the ROI-based MVPA, we also performed a searchlight MVPA, which enabled us to search locally informative voxels by running an MVPA with a moving small ROI (e.g., spherical cluster) across the whole brain^25^. To maximize the chance of detecting relevant signals, we took advantage of the higher signal-to-noise ratios and increased the BOLD signals available in ultra-high-field 7T fMRI^26^. We predicted that duration information would be decoded in the right IPL.

## Results

### Study overview

**Fig. 1:**
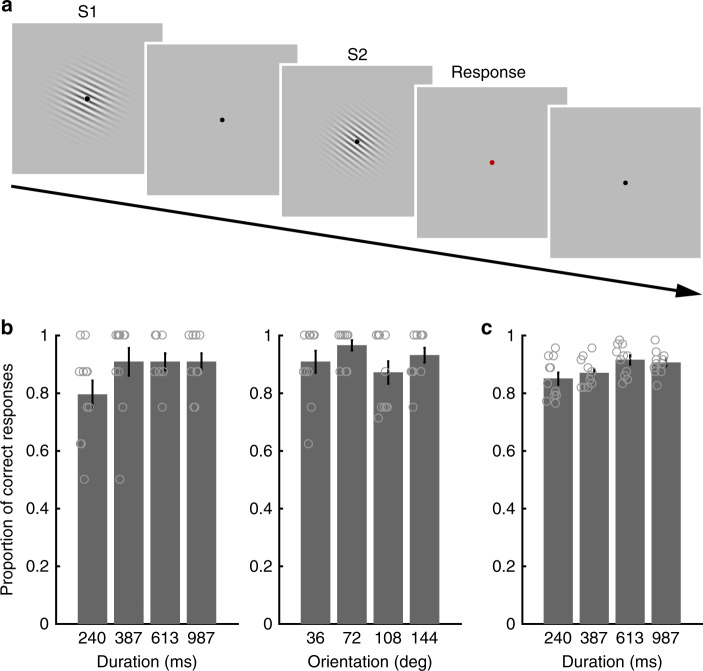
Stimulus sequence and task performances. **a** Stimulus sequence. In each trial, two visual stimuli (Gabor patches) and a response cue were sequentially presented. In the functional localizer scans, the participants performed the orientation discrimination task or the duration discrimination task according to the instruction cue presented in every 4 trials. In the main scans, the participants performed only the duration discrimination task. A response was made in every trial during the response period cued by a red fixation point. **b** Task performance in the localizer scans. Proportions of correct responses are plotted as a function of stimulus duration (left, duration task) and stimulus orientation (right, orientation task). **c** Task performance in the main scans. Proportions of correct responses in the duration task are plotted as a function of stimulus duration. Gray circles on the bar graphs indicate individual data. Error bars indicate standard errors of the mean

A group of healthy volunteers (*N* = 11) completed two runs of functional localizer scans and 18 runs of main scans separated into two imaging sessions. In each trial, in both types of scans, two visual stimuli (Gabor patches, S1 and S2) with varying durations and orientations were sequentially presented with a random interval that varied within a range of 4–5.2 s (Fig. 1a). S2 was followed by a response cue (red fixation point, duration 2 s). In the functional localizer scans, the participants performed both duration and orientation discrimination tasks, switching between tasks when instructed. In the main scans, the participants only performed a duration discrimination task.

Our analysis focused on the BOLD response at the offset of S1 because duration is determined by the offset of stimuli. First, using the data from localizer scans, we identified the brain regions that were activated during the duration or orientation tasks. The identified clusters were defined as ROIs for the subsequent ROI-based MVPA. In the ROI-based MVPA, data from the main scans were analysed by extracting multivoxel activity patterns within each ROI that had been defined by the previous step. The ROIs determined by the duration task were used to decode duration information and the ROIs determined by the orientation task were used for decoding orientation information in the main scans. To supplement the findings from the ROI-based MVPAs, a conventional general linear model (GLM) analysis and searchlight MVPA were also performed (see Methods section for the full details).

### Behavioral performance during functional localizer scans

The proportions of correct responses for the duration task in the localizer scans were 79.5 (mean) ± 16.1% (SD) (S1 duration = 240 ms), 90.9 ± 15.9% (384 ms), 90.9 ± 9.8% (614 ms), and 90.9 ± 9.8% (983 ms) (Fig. 1b, left). For the orientation task, the proportions of correct responses were 90.9 ± 12.6% (S1 orientation = 36 degrees), 96.6 ± 5.8% (72 degrees), 87.2 ± 12.9% (108 degrees) and 93.2 ± 8.6% (144 degrees) (Fig. 1b, right). One-way repeated measures analysis of variances (ANOVAs) for each task showed that the task performances were similar across durations in the duration task (main effect of duration, *F*_3,30_ = 2.604, *p* = 0.070, *η*^2^ = 0.207) and across orientations in the orientation task (main effect of orientation, *F*_3,30_ = 2.506, *p* = 0.078, *η*^2^ = 0.200). The proportions of overall correct responses were also comparable between duration and orientation tasks (88.1 ± 8.6% and 91.9 ± 7.7%, respectively; paired *t*-test, *t*_10_ = 1.039, *p* = 0.323, 95% confidence interval (CI) −0.043–0.119, Cohen’s *d* = 0.313).

### Behavioral performance in duration task during main scans

The proportions of correct responses for each duration were 85.1 (mean) ± 7.1% (SD) (S1 duration = 240 ms), 87.0 ± 4.9% (384 ms), 91.5 ± 5.4% (614 ms), and 90.6 ± 4.1% (983 ms) (Fig. 1c). Although the task performances were comparable across different durations in the localizer scans, a one-way repeated measures ANOVA in the main scans showed a significant main effect of duration (*F*_3,30_ = 4.824, *p* = 0.007, *η*^2^ = 0.325). However, post hoc pair-wise comparisons failed to show differences in the task performance between the combinations of S1 durations (*p* > 0.05, Bonferroni corrections for multiple comparisons). Notably, decoding performances reported in the following MVPA do not explain the differences in task performance.

### ROIs

**Fig. 2:**
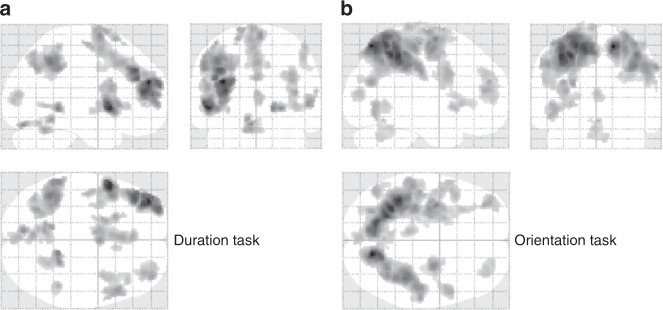
Results of the functional localizer scans. The brain areas activated at the offset of S1 in the **a** duration and **b** orientation discrimination tasks

To determine the ROIs for the following MVPAs, we initially determined the specific brain regions that showed positive responses at the offset of S1 in the localizer scans via a univariate analysis (i.e., general linear model; GLM). We identified 12 clusters in the duration task (Fig. 2a and Supplementary Table 1) and 11 clusters in the orientation task (Fig. 2b and Supplementary Table 2). Thresholded images for each identified cluster were used as ROIs in the following ROI-based MVPA.

### Frontal and parietal ROIs carry duration information

**Fig. 3:**
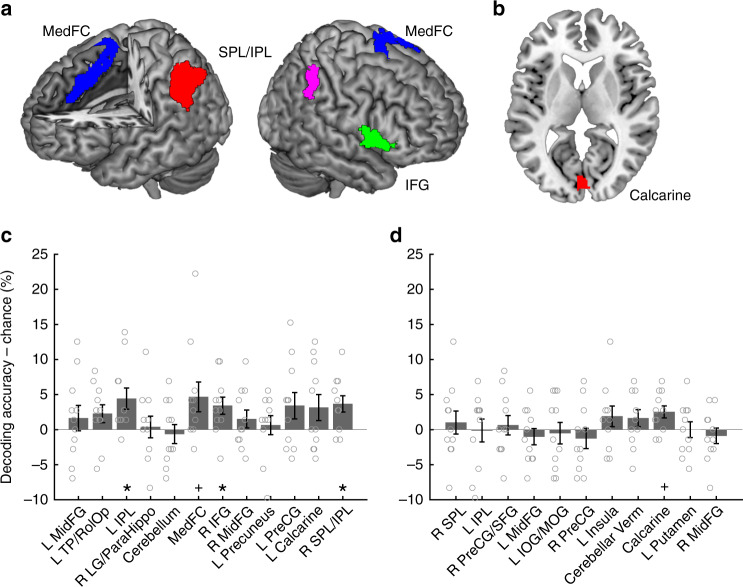
Results of the ROI-based multivariate pattern analysis. The ROIs that showed above-chance level classification accuracies for **a** stimulus duration and **b** orientation in the main scans (duration task). **a**, **c** Stimulus durations were decoded in the left IPL (red), the right SPL/IPL (pink), the right IFG (green) and, albeit less clearly, the MedFC (blue). **b**, **d** Stimulus orientations were decoded only in the calcarine ROI (red). The *y*-axes for **c** and **d** indicate decoding accuracy – chance level (i.e., 25 %). Gray circles on the bar graphs indicate individual data. Error bars indicate standard errors of the mean. *q < 0.05 FDR corrected, +*q* < 0.1 FDR corrected for multiple comparisons

To identify the brain regions that carry duration information, we performed a multiclass MVPA by extracting multi-voxel activity patterns in each ROI. We determined that three (of 12) ROIs, including the left IPL, right SPL/IPL and right IFG ROIs, showed above-chance level (i.e., >25%) classification performances (*q* < 0.05 false discovery rate (FDR) corrected) (left IPL, *t*_10_ = 2.931, *q* = 0.038, 95% CI = 1.060–7.779, Cohen’s *d* = 0.884; right SPL/IPL, *t*_10_ = 3.160, *q* = 0.038, 95% CI = 1.080–6.243, Cohen’s *d* = 0.953; right IFG, *t*_10_ = 2.797, *q* = 0.038, 95% CI = 0.693–6.125, Cohen’s *d* = 0.843). In addition, the medial frontal cortex (MedFC), which corresponds to the SMA, showed a trend (*q* < 0.1 FDR corrected) (*t*_10_ = 2.203, *q* = 0.078, 95% CI = −0.054–9.398, Cohen's *d* = 0.664) in an above-chance level decoding performance (Fig. 3a, c).

**Fig. 4:**
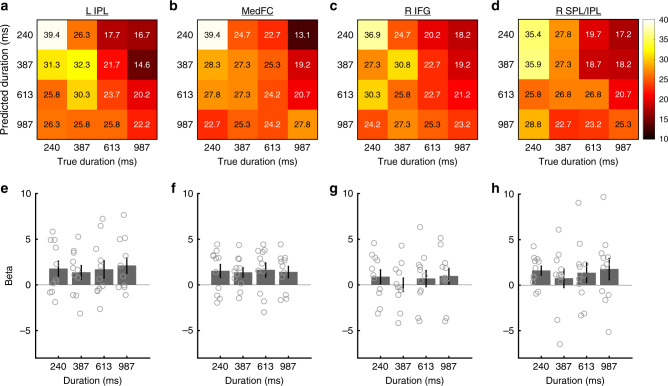
Confusion matrices and mean beta values in each ROI. **a**–**d** Proportions of predicted durations as a function of true (actual) stimulus durations, and **e**–**h** mean beta values for each duration condition in each ROI. The panel in each column corresponds to the data from the same ROI: **a**, **e** the left IPL, **b**, **f** MedFC, **c**, **g** right IFG, and **d**, **h** right SPL/IPL. In each cell in the confusion matrices (**a**–**d**), numerical values (%) are shown in addition to the color code. The color scale for the color code is shown on the right side of the top row. Gray circles on the bar graphs indicate individual data. Error bars in the bottom row (**e**–**h**) indicate standard errors of the mean

For a closer scrutiny of the classification performances, we examined the relationship between the predicted and true stimulus durations in the four ROIs (Fig. 4a–d) identified in the previous step. The confusion matrices indicated two characteristics that were common across the four ROIs. First, short S1 durations (i.e., 240 and 387 ms) tended to be predicted more accurately than longer durations (i.e., 613 and 987 ms). Second, when the classifier made incorrect predictions, the classifier tended to classify the duration as longer than the true duration. Thus, the classifier tended to overestimate the S1 duration.

As successful decoding by MVPA is generally associated with stimulus-dependent biases in the spatial activity pattern generated by neural populations tuned for the stimulus feature^24^, our finding of the above-chance level decoding performance in the frontal and parietal cortices may appear to be consistent with the idea of population coding of stimulus durations^8,14^. However, one alternative interpretation for the successful decoding performance is the existence of firing-rate coding of the stimulus duration, which may correspond to the accumulator in the clock-counter model of time perception^27^. The existence of an accumulator predicts greater overall BOLD responses as the stimulus duration increases^28–30^. To examine this possibility, the values of correlation coefficients (i.e., betas) for the S1 offset estimated by a GLM (refer to GLM analysis on main scans in the online Methods for details) were extracted and averaged across all voxels within each ROI (Fig. 4e–h). We subsequently performed a one-way repeated measures ANOVA for each ROI and determined that the overall activations were not different across different durations in these ROIs (left IPL, *F*_3,30_ = 1.230, *p* = 0.316, *η*^2^ = 0.110; right SPL/IPL, *F*_3,30_ = 0.973, *p* = 0.418, *η*^2^ = 0.089; right IFG, *F*_3,30_ = 2.526, *p* = 0.076, *η*^2^ = 0.202; MedFC, *F*_3,30_ = 0.293, *p* = 0.830, *η*^2^ = 0.029). The results were similar even when responses for S1 onset, rather than S1 offset, were examined (Supplementary Fig. 1). These results indicate that the above-chance level classification performances identified in the ROI-based MVPA are not explained by the overall difference in activations across different durations, which again supports the idea that duration information is represented by population coding and not by rate coding in these areas.

The general tendency of better decoding performances in relatively shorter durations (Fig. 4a–d) raises a concern that the better decoding performance may be associated with task performances, which were lower for shorter durations. Thus, the variability in the task difficulty across different duration conditions may have produced different multi-voxel activity patterns, which, in turn, contributed to the above-chance level decoding performances in the four ROIs. To address this potential concern, we examined whether the individual differences in the variability (i.e., standard deviations) of the task performances across different duration conditions predicted individuals’ decoding accuracies for each ROI. The Pearson’s correlation (one-tailed) showed that none of the four ROIs showed a correlation between these variables (left IPL, *r* = 0.066, *p* = 0.424, 95% CI = −0.474–1.000; Med FC, *r* = 0.053, *p* = 0.439, 95% CI = −0.484–1.000; right IFG, *r* = 0.340, *p* = 0.153, 95% CI = −0.223–1.000; right SPL/IPL, *r* = 0.189, *p* = 0.289, 95% CI = −0.372–1.000) (Supplementary Fig. 2), which suggests that the slight differences in task performances across different duration conditions do not account for the differences in the decoding performances for different durations.

**Fig. 5:**
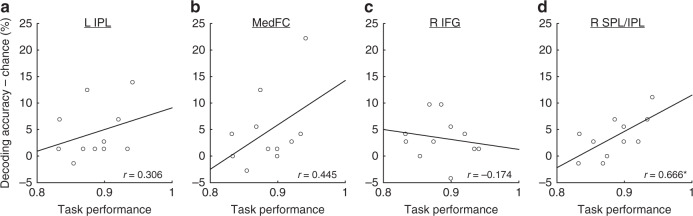
Correlations between the individual task performances and the decoding accuracies for each ROI. **a** The left IPL, **b** MedFC, **c** right IFG, and **d** right SPL/IPL. *p < 0.05 uncorrected

We subsequently examined whether individual differences in task performance predicted the decoding accuracy in these ROIs. The Pearson’s correlation coefficients (*r*) for each ROI were 0.306 (left IPL), 0.445 (MedFC), −0.174 (right IFG), and 0.666 (right SPL/IPL) (Fig. 5). The correlation in the right SPL/IPL was statistically significant (*p* = 0.013, 95% CI = 0.218–1.000; one-tailed) without correction for multiple comparisons and was marginally significant (*q* = 0.051) when an FDR correction was applied. The other ROIs were not statistically significant (left IPL, *q* = 0.240, 95% CI = −0.260–1.000; MedFC, *q* = 0.170, 95% CI = −0.103–1.000; right IFG, *q* = 0.695, −0.639–1.000; FDR corrected). This result suggests that the participants who showed better task performances exhibited more distinctive multi-voxel activity patterns in the right SPL/IPL for different stimulus durations.

### Occipital ROI carries orientation information

**Fig. 6:**
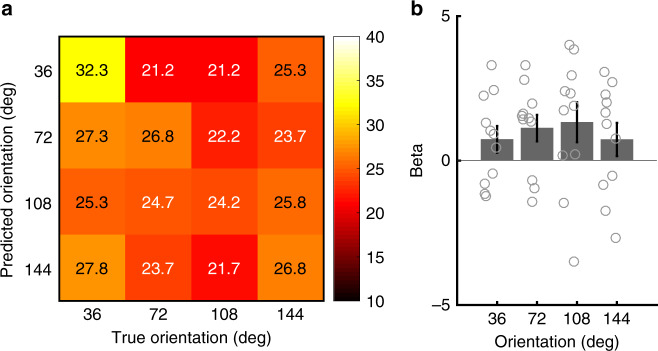
Confusion matrix and mean beta values of the occipital cortex. **a** Proportions of the predicted durations as a function of true (actual) stimulus orientations and **b** mean beta values for each duration condition. In each cell in the confusion matrix (**a**), numerical values (%) are shown in addition to the color code. The color scale of the color code is shown on the right. Gray circles on the bar graphs indicate individual data. Error bars indicate standard errors of the mean

Although our participants were paying attention to the stimulus durations to perform the task, our experimental design enabled us to determine which brain areas carried orientation information, as the manipulation of the orientation was orthogonal to the stimulus durations. We performed an ROI-based MVPA in the 11 ROIs identified in the orientation discrimination task in the localizer scans and determined that only the calcarine ROI showed a trend in the above-chance classification performance (*t*_10_ = 2.955, *q* = 0.079 FDR corrected, 95% CI = 0.621–4.429, Cohen’s *d* = 0.891) (Fig. 3b, d). The confusion matrix for this ROI is shown in Fig. 6a. A one-way repeated measures ANOVA showed that the differences in the overall activation levels across different orientation angles (Fig. 6b) were not significant (*F*_1.792,17.916_ = 1.732, *p* = 0.207 adjusted by Greenhouse-Geisser correction as the assumption of sphericity was violated, *η*^2^ = 0.148), which indicates that the trend in the above-chance decoding performance of orientation is not explained by the overall change in the activation level.

### Searchlight MVPA

To complement the results of the ROI-based MVPA, we performed a whole-brain searchlight MVPA to identify the brain areas that locally represent duration and orientation information.

### Duration information in frontoparietal and occipital regions

**Fig. 7:**
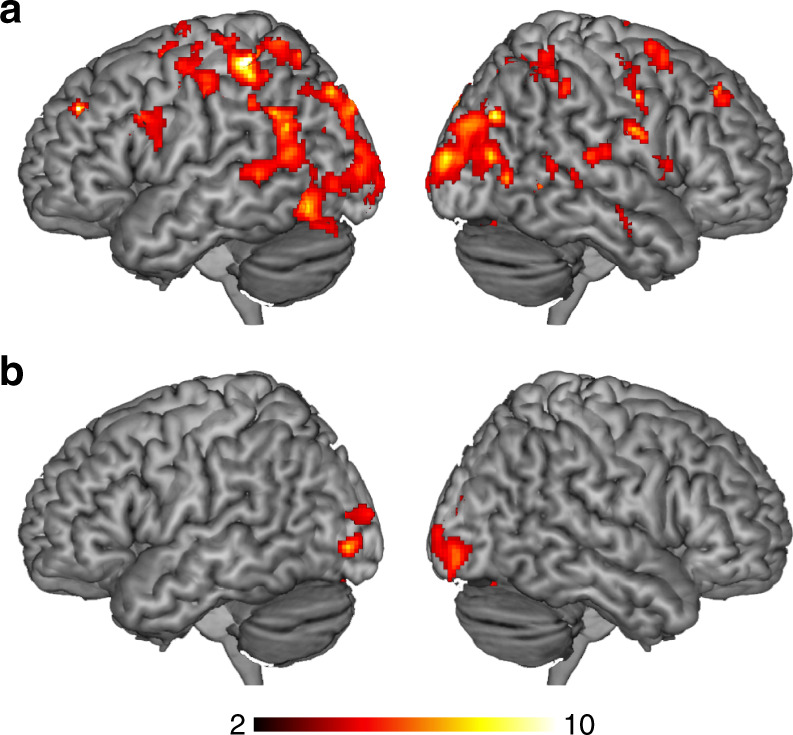
Results of the searchlight analyses at the group level. Brain regions that showed above-chance level (chance level = 25 %) classification accuracies for **a** stimulus durations and **b** orientations. The color scale indicates the *T*-values

**Fig. 8:**
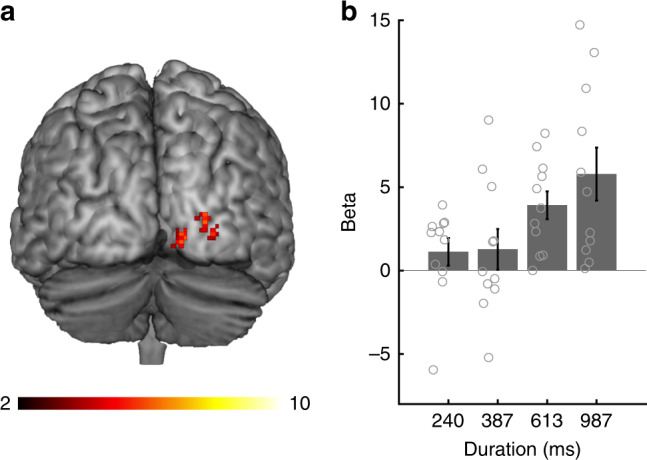
Monotonic increase in brain activity in the occipital cortex. **a** Brain regions that showed a monotonic increase in BOLD response according to the increase in stimulus durations. The color bar indicates the *T*-values. **b** The plot of beta values at the peak of the cluster in the occipital cortex (*x*, *y*, *z* = 26, −90, 2) for each stimulus duration. Gray circles on the bar graphs indicate individual data. Error bars indicate standard errors of the mean

The searchlight MVPA for the stimulus duration replicated the results of the ROI-based MVPA: local activity patterns in the frontoparietal regions, including the areas identified in the ROI-based MVPA, carried duration information (Fig. 7a and Supplementary Table 3; refer also to Supplementary Fig. 3a for the glass brain images). In addition to these areas, the searchlight MVPA identified a large cluster in the occipital cortex. As the above-chance level decoding performance in the occipital cortex could arise from a gradual increase in overall BOLD responses as a result of the increase in visual input, we performed another GLM analysis for the main scans. In the group-level whole-brain analysis, a linearly weighted parametric contrast identified 3 clusters in the occipital cortex that exhibited a gradual increase in BOLD responses with increased stimulus durations (Fig. 8 and Supplementary Table 4). This finding suggests that the above-chance level decoding performance in the occipital cortex may, at least in part, reflect the overall increase in the BOLD response according to the increase in stimulus durations.

### Orientation information in the visual cortex

The searchlight MVPA for decoding stimulus orientation showed that local activity patterns in the occipital cortex carried orientation information (Fig. 7b and Supplementary Table 5; refer also to Supplementary Fig. 3b for glass brain images). This finding is consistent with the results of the ROI-based approach that showed that orientation information was decoded from the calcarine ROI. None of the other areas showed above-chance decoding accuracy, which suggests that although duration information was widely distributed across the brain, orientation information was locally represented in the occipital cortex.

### Comparison of decoding accuracy between time and orientation

To examine whether any brain areas showed any difference in decoding performance, we directly compared the decoding accuracy for duration (Fig. 7a) and orientation (Fig. 7b). The results showed that multiple frontoparietal regions showed better decoding performance for duration than for orientation (Supplementary Fig. 5 and Supplementary Table 6). Most importantly, the right IPL, including the right supramarginal gyrus, showed a better decoding accuracy for duration than for orientation. This result, together with the results of the ROI-based analysis and searchlight analysis described above, suggests that the IPL exclusively carries duration information. In contrast, no brain areas showed a better decoding performance for orientation than for duration. The lack of superior decoding performance for orientation information in the occipital cortex is consistent with the finding in the searchlight analysis showing that the occipital cortex carried both orientation and duration information (see Fig. 7, Supplementary Tables 3 and 5).

## Discussion

Our study demonstrates that multiple brain areas, including the left IPL, the right SPL/IPL, the right IFG and, albeit less clearly, the MedFC, carry duration information. In addition, we determined that individual differences in decoding accuracy in the right parietal cortex correlated with task performances in duration judgments. These findings suggest that time information is distributed across frontoparietal regions and that the right parietal cortex in particular plays a crucial role in time estimation.

The above-chance level decoding performance in the right parietal cortex is consistent with the previous fMRI adaptation study that demonstrated duration selective neural adaptation occurred in the right IPL^14^. The present study provides additional support for the notion that the right IPL contains neural populations tuned for specific durations. Moreover, we determined that inter-individual differences in task performance predicted the decoding accuracy in the right SPL/IPL region. This finding indicates that individuals with better timing task performances showed more distinct multi-voxel activity patterns for different durations. In the past, numerous studies suggested the link between timing task performance and the right IPL. At a neuroanatomical level, individual differences in regional gray matter volume in the right IPL correlated with sensitivity in duration discrimination judgments^31^ (refer also to the Discussion in the ref. ^14^). The relevance of the right IPL in time estimation was also supported by transcranial magnetic stimulation (TMS) and brain lesion studies that indicated interference in right IPL activity impaired temporal processing^32–36^. Although these studies indicate a close link between the right IPL and timing performance, the functional difference in the right IPL that determines an individual’s time estimation ability is unclear. The correlation between the decoding accuracy and timing task performance suggests that the distinctiveness of neural response patterns for different durations in the right parietal cortex, which may arise from duration-tuned neural population activities^14^, determines an individual’s time estimation ability.

In the correlation analysis, our assumption was that the task performance of individuals would reflect their general ability to encode duration. However, one important caveat is that the correct/incorrect responses in each trial, which are reflected in the overall task performances, may, in fact, stem from the success and/or failure of encoding either S1 or S2, whereas decoding accuracy reflects the time representation of S1 alone. Given this potential mismatch of the source of variability in the two variables, further investigation is required to make a solid conclusion about whether individual differences in the time representation in the right IPL are predictive of duration discrimination performance. One possible approach to address this question is to examine whether, on a trial-by-trial basis, participants’ responses are predictable from decoded duration in the right IPL.

In addition to the right IPL, we determined that duration information was decoded in the left IPL. An explicit timing task often involves bilateral IPL activation^11^, whereas an implicit timing task in which temporal information is used for a non-temporal task goal (e.g., temporal orienting) has been associated with left IPL activity^37,38^. We speculate that the left IPL represents current duration information and uses that information to update prior knowledge of the statistical distribution of stimulus durations, which ultimately helps increase the precision of duration estimation^39^.

We also determined that several frontal areas, such as the right IFG and, albeit less clearly, the MedFC, carried duration information. This result is consistent with the previous meta-analysis of neuroimaging literature that indicated activation in these two areas was most frequently reported in studies that involved explicit perceptual and motor timing tasks^11^. Several recent studies have suggested that the right IFG plays a role in the decision-making process in temporal judgments. For example, a magnetoencephalography study reported that the right IFG was involved only when participants were actively engaged in temporal judgments, whereas this activity was absent when participants perceived the same stimuli without making temporal decisions^40^. Another line of research has suggested that the right IFG represents categorical information (i.e., shorter or longer) rather than metrical information of duration by showing that TMS over the right IFG impaired performance on a duration comparison task that requires categorical representation of time (i.e., shorter or longer) but not in a duration reproduction task that requires a metrical representation of time^34^. The idea of categorical time representation in the right IFG is also compatible with electrophysiological studies in monkeys indicating that the prefrontal cortex exhibited category-based activity during duration and spatial discrimination tasks^41,42^. From these previous studies, we speculate that time information represented in the right parietal cortex and IFG may be somewhat different: neural populations in the right parietal cortex are tuned to specific durations, whereas the right IFG is tuned to a temporal category (e.g., relatively short vs. relatively long). The above-chance level decoding accuracy in the right IFG may therefore be driven by multi-voxel activity patterns generated from category-tuned neural populations rather than duration-tuned neural populations.

In the present study, we reported that the BOLD response in the MedFC was comparable across different durations (Fig. 4f). This finding is consistent with the previous neuroimaging study showing a comparable BOLD response between 0.6 and 3.0 s^43^. In contrast, a recent fMRI study reported that the SMA, which overlapped with the MedFC in the present study, exhibited a greater BOLD response as the stimulus duration increased^28^, which is consistent with the notion that the climbing neural activity in the medial frontal region plays a role as temporal accumulator in the clock-counter model^27^. The reason for these mixed results is not clear and thus requires further investigation. Instead of the gradual increase in BOLD response, we found that, at the trend level, the multi-voxel activity pattern in the MedFC carries duration information. This finding suggests the importance of using the same set of experiments in the future to examine the relationship between the climbing neural activity in the frontal cortex and the offset-related response in the frontoparietal cortex.

Notably, the above-chance level decoding performances in the four frontoparietal ROIs (i.e., left IPL, right SPL/IPL, MedFC, and right IFG) may be explained by the slight difference in task difficulty between the different duration conditions (Fig. 1c). However, this alternative account is unlikely for two reasons. First, although the task difficulty account predicts greater BOLD responses for more difficult conditions, our GLM analysis failed to identify differences in the overall activity levels across different duration conditions in these ROIs (Fig. 4e–h). Moreover, the mean activity patterns did not follow the pattern of the mean task performances (Fig. 1c). Second, individual differences in the variance in task performances between different duration conditions were not correlated with the decoding accuracy (Supplementary Fig. 2), which suggests that the small variability in task difficulties between different duration conditions did not contribute to the decoding performances. Overall, these findings indicate that the above-chance level decoding performances in the frontoparietal ROIs reflect time representations and not task difficulty.

Our searchlight analysis showed that duration information was also decoded from local activity patterns in the occipital cortex. However, in contrast to other brain areas identified in the ROI-based MVPA, the visual cortex exhibited greater BOLD responses for longer durations, as shown in a previous study^28^. This finding suggests that in contrast to the frontal and parietal regions identified in the ROI-based MVPA, the above-chance decoding performance in the occipital cortex was driven by the modulation of overall BOLD responses for different stimulus durations rather than the multi-voxel activity patterns. However, it is not clear whether the increase in overall BOLD responses is a representation of time itself (i.e., temporal accumulation) or a simple reflection of greater (continuous) visual input for longer durations. One feasible approach to dissociate these possibilities is to examine whether the visual empty intervals (e.g., time intervals marked by two brief flashes), which lack continuous visual input, are decoded in the occipital cortex.

Given the finding of duration decoding in the occipital cortex in the searchlight analysis, one may question why duration information was not decoded in the left calcarine ROI in the ROI-based MVPA. One potential explanation for the insensitivity of the left calcarine ROI is that the BOLD response was not sensitive to the increase in visual input with increased stimulus durations because of saturation. This explanation is supported by data showing that the mean left calcarine ROI activity level was high at the short duration and that the level was comparable across durations (one-way repeated measures ANOVA; *F*_3,30_ = 0.645, *p* = 0.592, *η*^2^ = 0.061) (Supplementary Fig. 4). As we defined ROIs based on the GLM that highlighted the areas that showed significant activity regardless of duration (i.e., main effect of duration), it is possible that the voxels that showed a gradual increase in activity with increased durations were not captured (and thus not included in the calcarine ROI). This finding supports the notion that combining ROI-based and information-based (e.g., searchlight) approaches is beneficial in the exploration of neural representations with the MVPA technique.

The present study extends previous knowledge in the timing literature by showing that time information is widely distributed across the brain and that the right parietal cortex is associated with precise time estimation. The distributed time representation supports the multiple mechanism hypothesis, which predicts that multiple neural systems support timing function and these systems are flexibly engaged depending on task requirements^44^. Future studies should explore whether the right parietal cortex is associated with timing judgments regardless of the type of timing task (i.e., different modalities, duration ranges, motor timing, and implicit timing) as well as how the distributed, redundant time representations are weighted depending on the type of perception, cognition and action. Addressing these questions would help further understand how our brain generates a subjective experience of time and how humans optimize temporal behavior in a dynamically changing environment.

## Methods

### Participants

Eleven healthy, right-handed volunteers (six males and five females, mean age 23.7 years, SD 4.3 years, range 20–32) completed two sessions of fMRI experiments. The participants were students and academic staff recruited from the university community at Ecole Polytechnique Federale de Lausanne (EPFL) through an on-campus job search website and personal contacts. Prior to participating, all the participants provided written informed consent. The fMRI study protocol was approved by the local ethics committee (La Commission cantonale (VD) d'éthique de la recherche sur l'être humain, Protocol number 92/2012).

### Task and stimuli

Each participant completed two runs of functional localizer scans and 18 runs of main scans separated into two imaging sessions. The interval between the two sessions was between one and three days. In each session, one run of the functional localizer was followed by nine runs of the main scans. In each trial, in both types of scans, two visual stimuli (Gabor patches, S1 and S2) with varying durations and orientations were sequentially presented with a random interval that varied within a range of 4–5.2 s (in steps of 0.08 s). S2 was followed by a response cue (red fixation point, duration 2 s) (Fig. 1). A black fixation point was subsequently presented until the S1 for the next trial appeared, with a duration that varied within a range of 4–5.2 s (in steps of 0.08 s).

In the functional localizer scans, the participants performed both duration and orientation tasks, switching between tasks when instructed. For the duration task, the participants judged whether the duration of the second stimulus (S2) was shorter or longer than the first stimulus (S1). For the orientation task, the participants judged whether the orientation of S2 was rotated clockwise or counter-clockwise compared to S1. A black fixation point (duration 2 s) followed by an instruction cue (duration 1.5 s) and again a black fixation point (duration 4 s) were inserted in every 4 trials to indicate whether the task for the following 4 trials was a duration (Time) or orientation (Orientation) task. Each functional localizer scan contained 4 blocks of each task (16 trials each), and each run lasted 8 min 7 s. In the main scans, the participants performed the duration task only, no instruction cue was presented and no inter-block intervals were inserted. Each run of the main scans contained 16 trials and lasted 3 min 51 s.

The participants indicated their responses using two buttons on the button box (Current Designs, Philadelphia, Pennsylvania) held in their right hand. In the duration task, the participants were instructed to respond with their index or middle finger for shorter and longer responses, respectively. In the orientation task, the right index finger and middle finger corresponded to counter-clockwise and clockwise responses, respectively. The participants were instructed to respond as accurately as possible, with no emphasis on the response time. The participants were also instructed to fixate their eyes on the fixation point and ignore changes in the task-irrelevant stimulus features (i.e., orientation change in the duration task and duration change in the orientation task), as well as refrain from using a counting strategy to measure the durations in the duration task.

All stimuli were presented on a gray background. Psychtoolbox (http://psychtoolbox.org) implemented with MATLAB software (MathWorks, Natick, Massachusetts) was used to present the stimuli. The stimuli for S1 and S2 were sinusoidal Gabor patches (100 % contrast, spatial frequency of 1.9 cycles/degrees, Gaussian envelope SD of 2.2 degrees, diameter of ~9 degrees) with a circular hole (diameter 0.6 degrees) center of the patches presented at the center of the screen. A central fixation point (diameter 0.5 degrees) was always presented at the center of the screen. The S1 and S2 stimuli had two modulated dimensions: orientation and duration. The parameters for orientation and duration in S1 were varied at 4 levels each (orientation was 36, 72, 108, or 144 degrees from horizontal (0 degrees); duration was 240, 384, 614, or 983 ms) to create a total of 16 types of S1 stimuli (i.e., 4 × 4 combinations of orientation and duration parameters). Each stimulus type was presented only once in each run. In the orientation task (i.e., in the functional localizer scans), the orientation for S2 was a 10-degree rotation, in a clockwise or counter-clockwise direction, of the S1 orientation. The stimulus durations of S2 were randomly selected from the 4 duration parameters used for S1 (i.e., 240, 384, 614, or 983 ms). In the duration task (i.e., in both the functional localizer and main scans), the duration of S2 was determined based on a fixed Weber ratio of 0.5 with respect to the S1 duration (i.e., Weber ratio = (longer – shorter duration) / shorter duration). Thus, the Weber ratios for shorter and longer durations were equivalent (e.g., if S1 was 240 ms, S2 was 160 or 360 ms). The stimulus orientations of S2 in the duration task were randomly selected from 4 orientation parameters used for S1 (i.e., 36, 72, 108, or 144 degrees).

In the MRI scanner, visual stimuli were projected by an LCD projector onto a semi-transparent screen placed inside the scanner bore. The screen was viewed through a mirror mounted on the head coil.

### Behavioral data analysis

The proportions of correct responses were computed for the individuals’ behavioral data. For the behavioral data in the localizer scans, a paired sample *t*-test (*α* = 0.05) was performed to compare the overall accuracy between the duration and orientation tasks. Moreover, in both the localizer and main scans, one-way repeated measures ANOVAs (*α* = 0.05) were performed to determine whether there were statistically significant differences in the accuracy between the four different S1 parameters.

### MRI data acquisition

All MRI data were acquired with an actively shielded, head-only 7-Tesla MRI scanner (Siemens, Munich, Bavaria, Germany) equipped with a head gradient insert (AC84, 80 mT/m max gradient strength; 350 mT/m/s slew rate) and 32-channel receive coil with a tight transmit sleeve (Nova Medical, Wilmington, Massachusetts). For each individual, 3754 volumes of functional MRI data (356 volumes × 2 runs for functional localizer scans and 169 volumes × 18 runs for main scans) were collected using the 3D-EPI-CAIPI sequence^45^ with the following parameters: nominal spatial resolution = 2.0 mm isotropic, volume acquisition time = 1368 ms, flip angle = 14 degrees, repetition time (TR) = 57 ms, echo time (TE) = 26 ms and bandwidth = 2774 Hz/Px. The matrix size was 106 × 88 × 72, which resulted in a field of view of 210 (AP) × 175 (RL) × 144 (FH) mm. An undersampling factor 3 and CAIPIRINHA shift 1 were used. Slices were oriented transversally with the phase-encoding direction left-right. For the GRAPPA reconstruction, 42 × 45 reference lines were acquired. High-resolution whole-brain MR images were also obtained using the MP2RAGE sequence^46^ (voxel size = 1.0 × 1.0 × 1.0 mm, matrix size 256 × 256 × 176, TI_1_/TI_2_ = 750/2350 ms, *α*_1_/*α*_2_ = 4/5 degrees, TR_MP2RAGE_/TR/TE = 5500/6.5/2.84 ms). Data from the main scans were shared with another study (https://www.biorxiv.org/content/early/2018/08/24/399857).

### Pre-processing of fMRI data

Pre-processing and GLM analyses of the localizer fMRI data were performed using statistical parametric mapping software (SPM12; http://www.fil.ion.ucl.ac.uk/spm/) implemented in MATLAB. The functional localizer data were realigned and normalized in Montreal Neurological Institute (MNI) space using the unified segmentation and normalization procedure provided in SPM12. The normalized fMRI data were subsequently smoothed in three dimensions using a 6-mm full-width-at-half-maximum Gaussian kernel.

For the main scan data, we pre-processed the data in two different ways for two different types of analysis: ROI-based MVPA and searchlight MVPA. For the ROI-based MVPA, the fMRI data were realigned and normalized in MNI space using the unified segmentation and normalization procedure provided in SPM12. Smoothing was not performed to retain the spatial specificity of the BOLD response. For the searchlight analysis, we performed only realignment and reslicing. Normalization to the MNI space was only performed after performing the searchlight MVPA (refer to the Searchlight MVPA section for additional details).

### GLM analysis for functional localizer scans

To identify the brain areas that responded to the offset of S1, pre-processed data of the localizer scans were analysed with a GLM. The GLM modeled offsets of S1, onsets of S2, button responses, and onsets of the instruction cue. The S1 and S2 in the time task and orientation task were included as separate regressors. For S1, we modeled the offsets of S1 but not the onsets because duration information becomes available only at the offset of stimuli. For the offsets of S1, 4 stimulus parameters for each task were modeled separately to estimate values of the regression coefficient (i.e., beta) independently for each parameter. Motion parameters estimated in the realignment procedure (refer to the pre-processing of fMRI data section) were also included in the GLM to regress the potential motion-induced signal fluctuations. In total, 12 regressors of interest (i.e., 12 regressors (2 (task) × 4 (S1-offset) + 2 (task) × 1 (S2-onset) + 1 (button response) + 1 instruction cue), duration = 0) and 6 regressors of no-interest (i.e., motion parameters) were set for each run. Each regressor of interest was convolved by a canonical haemodynamic response (HRF) function. The model of each participant was high-pass filtered (128 s), and a baseline was included to capture session effects.

The individual subject data of the localizer scans were incorporated into a group-level analysis using a random effect model. This group-level analysis was aimed at identifying S1-offset-related activation. First, the images of parameter estimates (contrast images) of the mean S1-offset responses for each task were obtained via individual analysis. A full factorial analysis was subsequently performed to obtain population inference for the S1-offset responses in time and orientation tasks separately. We used a relatively liberal threshold of *p* < 0.01 voxel-level uncorrected (cluster size *k* > 140 voxels) to create ROIs for the following MVPAs. The anatomical label of each ROI was determined based on the locations of peak coordinates of the clusters. Two anatomical labels were assigned to some ROIs (e.g., SPL/IPL), since multiple peaks located in different anatomical areas were identified in the cluster.

### GLM analysis for main scans

The pre-processed data of the main scans (i.e., pre-processed data for both ROI-based MVPA and searchlight MVPA) were modeled by GLMs to obtain beta images that were used in the following MVPA. For individual data, we applied two design matrices, the Time design matrix and the Orientation design matrix, to estimate the beta values for each duration and orientation parameter of S1. Both matrices modeled the offsets of S1, the onsets of S2, button responses, and onsets of the instruction cue; however, the Time design matrix and the Orientation design matrix differed in modeling the S1 offsets. In the Time design matrix, 4 duration parameters were independently modeled regardless of the stimulus orientation. Similarly, in the Orientation design matrix, 4 orientation parameters were independently modeled regardless of the stimulus duration. Motion parameters estimated in the realignment procedure (refer to Pre-processing of fMRI data) were also included in the model to regress the potential motion-induced signal fluctuations. In total, 6 regressors of interest (i.e., 4 (S1-offset) + 1 (S2-onset) + 1 (button response), duration = 0) and 6 regressors of no-interest (i.e., motion parameters) were set for each run of each matrix. Each regressor of interest was convolved by a canonical haemodynamic response (HRF) function. The model of each participant was high-pass filtered (128 s), and a baseline was included to capture session effects.

### MVPA for main scans

The MVPAs were performed with The Decoding Toolbox v3.52^47^ implemented in MATLAB. The images of the beta values for each level of the S1 parameter (i.e., 4 levels of S1-duration/orientation) estimated by the Time design matrix and the Orientation design matrix (i.e., a total of 144 beta images for each ROI-based and searchlight MVPA; 4 images per run × 18 runs × 2 design matrices) were incorporated into the standard ROI-based and searchlight MVPA pipeline.

### ROI-based MVPA

To determine the specific ROI that carried duration and orientation information, we performed multiclass ROI-based MVPAs. The multiclass ROI-based MVPAs for decoding duration and orientation information were performed separately but with the same procedure. A linear support vector machine classifier was trained using samples of 17 runs, and classification was performed for the samples of the remaining run to evaluate the performance of the classifier. This leave-one-run-out cross-validation procedure was repeated for all combinations of runs in the main scans. The decoding accuracy was computed for each individual in each ROI. The summary of the classification performance was subsequently assessed in the group-level analysis to determine whether the classification performance was above the chance level (i.e., 25%). The values of [decoding accuracy – chance (%)] were tested for each ROI using one-sample *t*-tests with a statistical threshold of *q* < 0.05 FDR-corrected.

### Searchlight MVPA

To provide complementary information to the ROI-based MVPA, we performed a searchlight MVPA, which enabled us to search locally informative voxels by running an MVPA with a moving small ROI (e.g., spherical cluster) across the whole brain^25^. Beta images for each level of S1 parameters (i.e., 4 levels of S1-duration/orientation) estimated by the Time design matrix and the Orientation design matrix were incorporated into the searchlight MVPA (i.e., a total of 144 beta images, 4 images per run × 18 runs × 2 design matrices). To identify the locally informative areas, a spherical cluster (radius = 4 voxels, cluster size ~ 268 voxels) was created for a whole-brain search. The training of a linear support vector machine classifier and the evaluation of classification performance were performed via a leave-one-run-out cross-validation procedure. The accuracy maps (i.e., decoding accuracy – chance level) obtained by this procedure were subsequently normalized against the MNI space and smoothed using a 6-mm full-width-at-half-maximum Gaussian kernel. The smoothed images were assessed in the group-level random effect analysis for group inferences using one-sample *t*-tests. A direct comparison of decoding performances between duration and orientation was performed using a paired *t*-test. The statistical threshold was set at a *p* < 0.001 voxel-level uncorrected and *q* < 0.05 cluster-level FDR corrected.

### Data visualization

For visualization of ROI-masks and the results of ROI-based MVPA and searchlight MVPA, cluster images were superimposed on a standard brain template using MRIcron software (http://people.cas.sc.edu/rorden/mricron/index.html).

## Supplementary information

Supplementary Information

Supplementary Data 1

Description of Additional Supplementary Files

## Data Availability

The datasets generated during and/or analysed during the current study are available from the corresponding author on reasonable request. All source data underlying the plots presented in the figures are available in Supplementary Data 1.

## References

[1] Buhusi CV, Meck WH (2005). What makes us tick? Functional and neural mechanisms of interval timing. Nat. Rev. Neurosci..

[2] Mauk MD, Buonomano DV (2004). The neural basis of temporal processing. Annu. Rev. Neurosci..

[3] Magyari L, De Ruiter JP, Levinson SC (2017). Temporal preparation for speaking in question-answer sequences. Front. Psychol..

[4] Ivry RB (1996). The representation of temporal information in perception and motor control. Curr. Opin. Neurobiol..

[5] Treisman M (1963). Temporal discrimination and the indifference interval: Implications for a model of the” internal clock”. Psychol. Monogr..

[6] Buonomano DV, Maass W (2009). State-dependent computations: spatiotemporal processing in cortical networks. Nat. Rev. Neurosci..

[7] Buonomano DV, Laje R (2010). Population clocks: motor timing with neural dynamics. Trends Cogn. Sci..

[8] Heron J (2012). Duration channels mediate human time perception. Proc. Biol. Sci..

[9] Ivry RB, Schlerf JE (2008). Dedicated and intrinsic models of time perception. Trends Cogn. Sci..

[10] Merchant H, Harrington DL, Meck WH (2013). Neural basis of the perception and estimation of time. Annu. Rev. Neurosci..

[11] Wiener M, Turkeltaub P, Coslett HB (2010). The image of time: a voxel-wise meta-analysis. Neuroimage.

[12] Church RM (1984). Properties of the internal clock. Ann. N. Y. Acad. Sci..

[13] Gibbon J, Church RM, Meck WH (1984). Scalar timing in memory. Ann. N. Y. Acad. Sci..

[14] Hayashi MJ (2015). Time adaptation shows duration selectivity in the human parietal cortex. PLoS Biol..

[15] Grill-Spector K, Henson R, Martin A (2006). Repetition and the brain: neural models of stimulus-specific effects. Trends Cogn. Sci..

[16] Krekelberg B, Boynton GM, van Wezel RJ (2006). Adaptation: from single cells to BOLD signals. Trends Neurosci..

[17] Fulcher, C., McGraw, P. V., Roach, N. W., Whitaker, D. & Heron, J. Object size determines the spatial spread of visual time. *Proc. Biol. Sci.* **283**, 20161024 (2016). 10.1098/rspb.2016.1024 PMC4971211 27466452

[18] Li B, Yuan X, Chen Y, Liu P, Huang X (2015). Visual duration aftereffect is position invariant. Front. Psychol..

[19] Li B, Xiao L, Yin H, Liu P, Huang X (2017). Duration aftereffect depends on the duration of adaptation. Front. Psychol..

[20] Maarseveen J, Hogendoorn H, Verstraten FA, Paffen CL (2017). An investigation of the spatial selectivity of the duration after-effect. Vision. Res.

[21] Shima S, Murai Y, Hashimoto Y, Yotsumoto Y (2016). Duration adaptation occurs across the sub- and supra-second systems. Front Psychol..

[22] Barron, H. C., Garvert, M. M. & Behrens, T. E. Repetition suppression: a means to index neural representations using BOLD. *Philos. Trans. R. Soc. Lond. B* **371**, 20150355 (2016). 10.1098/rstb.2015.0355 PMC5003856 27574308

[23] Haynes JD (2015). A primer on pattern-based approaches to fMRI: principles, pitfalls, and perspectives. Neuron.

[24] Norman KA, Polyn SM, Detre GJ, Haxby JV (2006). Beyond mind-reading: multi-voxel pattern analysis of fMRI data. Trends Cogn. Sci..

[25] Kriegeskorte N, Goebel R, Bandettini P (2006). Information-based functional brain mapping. Proc. Natl Acad. Sci. USA.

[26] van der Zwaag W (2009). fMRI at 1.5, 3 and 7 T: characterising BOLD signal changes. Neuroimage.

[27] Macar F, Vidal F (2009). Timing processes: an outline of behavioural and neural indices not systematically considered in timing models. Can. J. Exp. Psychol..

[28] Coull JT, Charras P, Donadieu M, Droit-Volet S, Vidal F (2015). SMA selectively codes the active accumulation of temporal, not spatial, magnitude. J. Cogn. Neurosci..

[29] Macar F, Vidal F, Casini L (1999). The supplementary motor area in motor and sensory timing: evidence from slow brain potential changes. Exp. Brain Res..

[30] Wencil EB, Coslett HB, Aguirre GK, Chatterjee A (2010). Carving the clock at its component joints: neural bases for interval timing. J. Neurophysiol..

[31] Hayashi MJ, Kantele M, Walsh V, Carlson S, Kanai R (2014). Dissociable neuroanatomical correlates of subsecond and suprasecond time perception. J. Cogn. Neurosci..

[32] Bueti D, Bahrami B, Walsh V (2008). Sensory and association cortex in time perception. J. Cogn. Neurosci..

[33] Harrington DL, Haaland KY, Knight RT (1998). Cortical networks underlying mechanisms of time perception. J. Neurosci..

[34] Hayashi MJ (2013). Interaction of numerosity and time in prefrontal and parietal cortex. J. Neurosci..

[35] Wiener M, Hamilton R, Turkeltaub P, Matell MS, Coslett HB (2010). Fast forward: supramarginal gyrus stimulation alters time measurement. J. Cogn. Neurosci..

[36] Wiener M (2012). Parietal influence on temporal encoding indexed by simultaneous transcranial magnetic stimulation and electroencephalography. J. Neurosci..

[37] Coull JT, Davranche K, Nazarian B, Vidal F (2013). Functional anatomy of timing differs for production versus prediction of time intervals. Neuropsychologia.

[38] Wiener M, Turkeltaub PE, Coslett HB (2010). Implicit timing activates the left inferior parietal cortex. Neuropsychologia.

[39] Shi Z, Church RM, Meck WH (2013). Bayesian optimization of time perception. Trends Cogn. Sci..

[40] Hironaga N (2017). Spatiotemporal brain dynamics of auditory temporal assimilation. Sci. Rep..

[41] Genovesio A, Tsujimoto S, Wise SP (2009). Feature- and order-based timing representations in the frontal cortex. Neuron.

[42] Genovesio A, Tsujimoto S, Wise SP (2011). Prefrontal cortex activity during the discrimination of relative distance. J. Neurosci..

[43] Lewis PA, Miall RC (2003). Brain activation patterns during measurement of sub- and supra-second intervals. Neuropsychologia.

[44] Wiener M, Matell MS, Coslett HB (2011). Multiple mechanisms for temporal processing. Front. Integr. Neurosci..

[45] Narsude M, Gallichan D, van der Zwaag W, Gruetter R, Marques JP (2016). Three-dimensional echo planar imaging with controlled aliasing: a sequence for high temporal resolution functional MRI. Magn. Reson. Med..

[46] Marques JP (2010). MP2RAGE, a self bias-field corrected sequence for improved segmentation and T1-mapping at high field. Neuroimage.

[47] Hebart MN, Görgen K, Haynes JD (2014). The decoding toolbox (TDT): a versatile software package for multivariate analyses of functional imaging data. Front. Neuroinform..

