# Author Correction: Representations of time in human frontoparietal cortex

**DOI:** 10.1038/s42003-022-04053-w

**Published:** 2022-10-10

**Authors:** Masamichi J. Hayashi, Wietske van der Zwaag, Domenica Bueti, Ryota Kanai

**Affiliations:** 1 Global Center for Medical Engineering and Informatics, Osaka University, Suita, 565-0871 Japan; 2 School of Psychology, University of Sussex, Brighton, BN1 9QH UK; 3 Center for Biomedical Imaging, Ecole Polytechnique Fédérale de Lausanne, Lausanne, CH-1015 Switzerland; 4 Spinoza Centre for Neuroimaging, Amsterdam, 1105BK The Netherlands; 5 International School for Advanced Studies, Trieste, 34136 Italy; 6 Araya Inc., Tokyo, 105-0003 Japan; 7 Sackler Centre for Consciousness Science, University of Sussex, Brighton, BN1 9QH UK

Correction to: *Communications Biology* 10.1038/s42003-018-0243-z, published online 21 December 2018.

The original version of this Article contained errors in the text.

The Methods subsection on Task and stimuli, fourth paragraph, incorrectly stated: “In the duration task (i.e., in both the functional localizer and main scans), the duration of S2 was determined based on a fixed Weber ratio of ~0.4 relative to the S1 duration (i.e., Weber ratio = shorter duration / (longer + shorter duration)).” The text should read, “In the duration task (i.e., in both the functional localizer and main scans), the duration of S2 was determined based on a fixed Weber ratio of 0.5 with respect to the S1 duration (i.e., Weber ratio = (longer – shorter duration) / shorter duration).”

The Methods subsection on Pre-processing of fMRI data, first paragraph, incorrectly stated: “The functional localizer data were realigned and normalized against the Montreal Neurological Institute (MNI) stereotactic space using diffeomorphic anatomical registration through exponentiated lie algebra (DARTEL) algorithms in SPM12.” The authors normalized using a unified segmentation and normalization procedure, the standard normalization procedure in SPM12, instead of an optional procedure DARTEL for spatial normalization. The text should read, “The functional localizer data were realigned and normalized in Montreal Neurological Institute (MNI) space using the unified segmentation and normalization procedure provided in SPM12.”

Further, the Methods subsection on Pre-processing of fMRI, second paragraph, incorrectly stated: “For the ROI-based MVPA, the fMRI data were realigned and normalized to the MNI space using DARTEL.” The text should read, “For the ROI-based MVPA, the fMRI data were realigned and normalized in MNI space using the unified segmentation and normalization procedure provided in SPM12.”

This has now been corrected in the PDF and HTML versions of the Article.

