# Supplementary material for: Representations of time in human frontoparietal cortex
Source: Commun Biol. 2018 Dec 21;1:233. doi: 10.1038/s42003-018-0243-z (PMC6303258; doi:10.1038/s42003-018-0243-z)
Supplement: Supplementary file 1 — Supplementary Information [file 42003_2018_243_MOESM1_ESM.pdf]

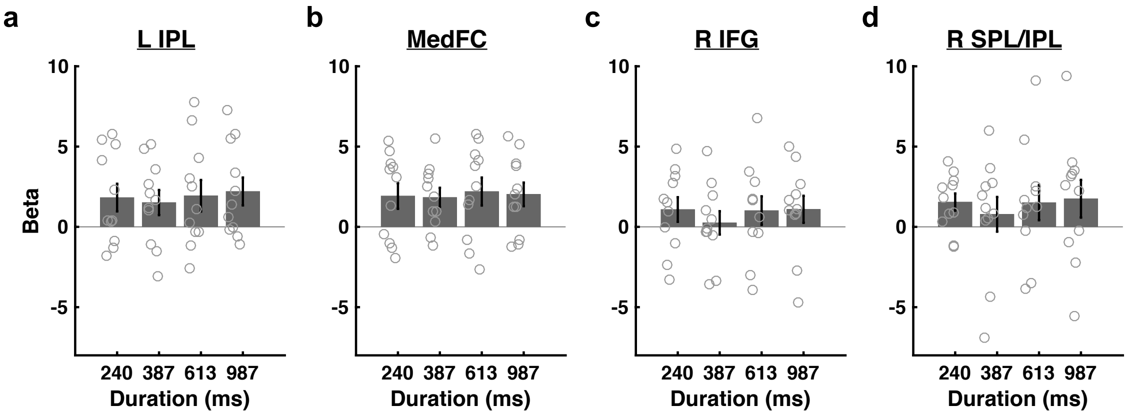

Supplementary Fig. 1

Mean beta values in each ROI.

(a-d) The mean beta values for each duration condition in the (a) left IPL, (b) MedFC, (c) right IFG, and (d) right SPL/IPL. One-way repeated measures ANOVAs with within-factor of test duration showed that overall activation levels were equivalent across durations in all ROIs (left IPL, $F_{3,30} = 1.230$, $p = 0.316$, $\eta^2 = 0.110$; MedFC, $F_{3,30} = 0.506$, $p = 0.681$, $\eta^2 = 0.048$; right IFG, $F_{3,30} = 2.246$, $p = 0.103$, $\eta^2 = 0.183$; right SPL/IPL, $F_{3,30} = 0.938$, $p = 0.435$, $\eta^2 = 0.086$). Gray circles on the bar graphs indicate individual data. Error bars indicate standard errors of the mean.

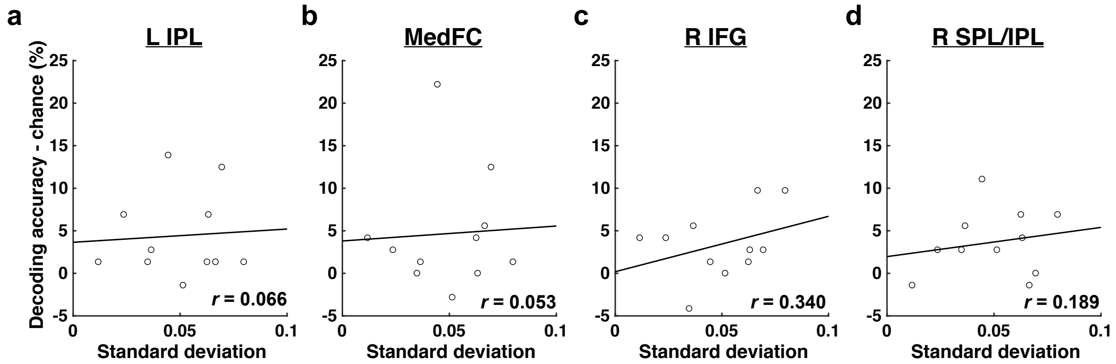

Supplementary Fig. 2

Correlations between individual differences in standard deviation in the task performances across different duration conditions and decoding accuracies for each ROI.

(**a**) Left IPL, (**b**) MedFC, (**c**) right IFG, and (**d**) right SPL/IPL.

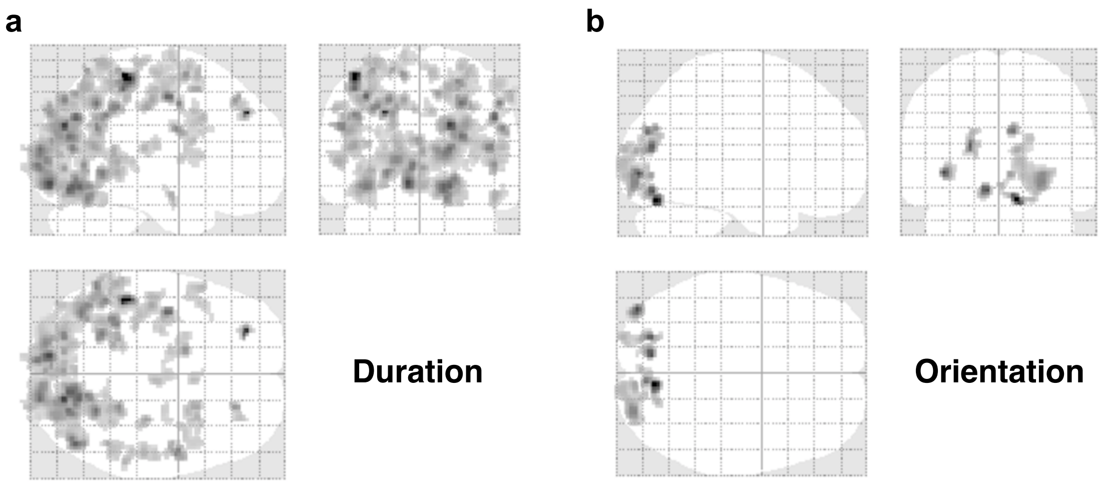

Supplementary Fig. 3

Results of the searchlight analyses at the group level.

Brain regions that showed above-chance level (chance level = 25 %) classification accuracies for (**a**) stimulus durations and (**b**) orientations. The data presented here are identical to the data in Fig. 5 but are rendered on glass brain images.

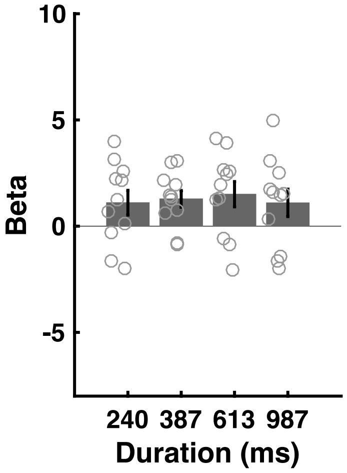

Supplementary Fig. 4

Mean beta values in the left calcarine ROI.

The mean beta values for each duration condition. Gray circles on the bar graphs indicate individual data. Error bars indicate standard errors of the mean.

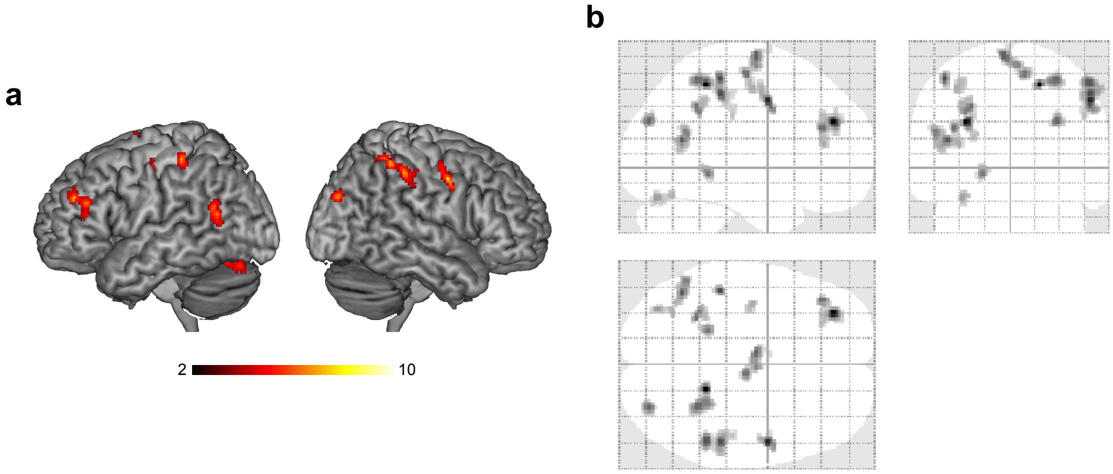

Supplementary Fig. 5

Brain areas that showed better decoding performances for duration than for orientation. The results were rendered on the standard (**a**) volume images and (**b**) glass brain images. The colour scale indicates the T-values.

Supplementary Table 1

Activated clusters as the effect of S1 in the duration task

			MNI coordinates			
Cluster size	Location	Side	x	y	z	Z-value
1381	MidFG	L	-36	50	22	5.38
926	TP/RolOp	L	-52	10	-4	5.22
1852	IPL	L	-42	-52	44	4.31
212	LG/ParaHippo	R	14	-44	-4	4.20
330	Cerebellum	–	-6	-80	-20	3.98
1341	MedFC	–	2	0	70	3.92
439	IFG	R	52	18	2	3.82
492	MidFG	R	38	54	12	3.63
172	Precuneus	L	-2	-70	50	3.52
215	PreCG	L	-44	-12	60	3.44
157	Calcarine	L	0	-84	10	3.33
160	SPL/IPL	R	48	-42	58	3.31

MidFG, middle frontal gyrus; TP, temporal pole; RolOP, rolandic operculum; IPL, inferior parietal lobule; LG, lingual gyrus; ParaHippo, para hippocampal gyrus; MedFC, medial frontal cortex; IFG, inferior frontal gyrus; PreCG, precentral gyrus; SPL, superior parietal lobule; IPL, inferior parietal lobule.

Supplementary Table 2

Activated clusters as the effect of S1 in the orientation task

			MNI coordinates			
Cluster size	Location	Side	x	y	z	Z-value
2929	SPL	R	16	-72	58	5.95
7802	IPL	L	-28	-52	48	5.68
609	PreCG/SFG	R	28	-8	56	4.37
382	MidFG	L	-36	50	22	3.92
336	IOG/MOG	L	-46	-70	-6	3.78
345	PreCG	R	52	8	30	3.77
142	Insula	L	-42	14	-4	3.55
241	Cerebellar Verm	–	0	-60	-16	3.51
140	Calcarine	–	0	-84	10	3.46
151	Putamen	L	-28	20	0	3.37
218	MidFG	R	34	46	30	3.24

SPL, superior parietal lobule; IPL, inferior parietal lobule; PreCG, precentral gyrus; SFG, superior frontal gyrus; MidFG, middle frontal gyrus; IOG, inferior occipital gyrus; MOD, middle occipital gyrus; Cerebellar Verm, cerebellar vermis.

Supplementary Table 3

Results of the searchlight MVPA for the stimulus duration

			MNI coordinates			
Cluster size	Location	Side	x	y	z	Z-value
369	PostCG/IPL	L	-46	-38	60	5.29
45	MidFG	L	-26	44	36	4.83
5801	SOG/Calcarine	–	18	-80	28	4.80
56	AG	R	48	-72	32	4.57
405	PreCG/PostCG	L	-38	-10	46	4.54
61	PreCG	R	52	-2	42	4.29
770	IPL/AG	L	-32	-46	40	4.27
69	PostCG/PreCG	R	54	-4	26	4.22
262	MedFC	–	-14	-2	65	4.11
55	SFG/MidFG	R	24	36	44	4.05
200	SPL	L	-34	-64	64	4.01
138	SFG/MidFG	R	24	10	52	3.91
119	MTG	R	48	-50	-2	3.87
100	STG/RolOp	R	54	-26	12	3.78
169	IFG	L	-54	14	32	3.76
45	IPL	R	60	-38	46	3.73
43	MCC	R	6	8	36	3.65
167	Insula	R	32	8	18	3.62
63	MOG	L	-18	-74	18	3.61
63	IPL	R	52	-46	54	3.60
46	SFG/PreCG	R	26	-12	58	3.40

PostCG, postcentral gyrus; IPL, inferior parietal lobule; MidFG, middle frontal gyrus; SOG, superior occipital gyrus; AG, angular gyrus; PreCG, precentral gyrus; MedFC, medial frontal cortex; SFG, superior frontal gyrus; SPL, superior parietal lobule; MTG, middle temporal gyrus; STG, superior temporal gyrus; RolOp, rolandic operculum; IFG, inferior frontal gyrus; MCC, middle cingulate cortex; MOG, middle occipital gyrus. Note: Two clusters that appeared in the white matter were omitted from this table.

Supplementary Table 4

Brain regions that showed a monotonic increase in activity as a function of stimulus duration

			MNI coordinates			
Cluster size	Location	Side	x	y	z	Z-value
13	MOG	R	26	-90	2	4.45
23	Calcarine/LG	R	16	-94	-6	4.42
8	IOG	R	30	-88	-4	4.08

MOG, middle occipital gyrus; LG, lingual gyrus; IOG, inferior occipital gyrus.

Supplementary Table 5

Results of the searchlight MVPA for stimulus orientation

			MNI coordinates			
Cluster size	Location	Side	x	y	z	Z-value
112	Cerebellum/LG	–	10	-76	-20	4.73
90	MOG	L	-40	-88	-2	4.27
80	Cerebellum/LG	L	-10	-78	-14	4.23
181	MOG	L	-22	-98	14	4.15
53	Cuneus	R	8	-82	26	4.01
587	IOG/Calcarine	R	28	-90	-8	3.88

LG, lingual gyrus; MOG, middle occipital gyrus; IOG, inferior occipital gyrus.

Supplementary Table 6

Comparison of searchlight decoding accuracies for duration and for orientation

			MNI coordinates			
Cluster size	Location	Side	x	y	z	Z-value
Duration decoding > Orientation decoding						
152	SPL	R	18	-44	52	4.51
183	MFG/IFG	L	-32	42	28	4.45
78	PreCG	R	52	-2	42	4.31
41	PostCG	L	-46	-34	56	4.12
201	SMG/IPL	R	52	-34	46	4.10
134	MTG	L	-44	-60	16	4.02
131	PostMedFC	–	-6	-10	70	4.01
51	MOG	R	30	-82	28	3.96
59	IPL	L	-28	-50	38	3.90
39	ParaHipp	L	-20	-42	-6	3.86
42	Cerebellum	L	-34	-76	-22	3.59
41	PreCG	L	-38	-12	50	3.52
Duration decoding < Orientation decoding						
No suprathreshold clusters						

SPL, superior parietal lobule; MFG, middle frontal gyrus; IFG, inferior frontal gyrus; PreCG, precentral gyrus; PostCG, postcentral gyrus; SMG, supramarginal gyrus; IPL, inferior parietal lobule; MTG, middle temporal gyrus; PostMedFC, posterior-medial frontal cortex; MOG, middle occipital gyrus; ParaHipp, parahippocampal gyrus.
