# Supplementary material for: Representations of time in human frontoparietal cortex
Source: Commun Biol. 2018 Dec 21;1:233. doi: 10.1038/s42003-018-0243-z (PMC6303258; doi:10.1038/s42003-018-0243-z)
Supplement: Supplementary file 3 — Description of Additional Supplementary Files [file 42003_2018_243_MOESM3_ESM.docx]

Description of Supplementary Data 1

**Supplementary Data 1**

Data used to generate the graphs shown in figures 1, 3, 4, 5, 6 and 8 and Supplementary figures 1, 2 and 4.
